# Towards Personalized Cardiology: Multi-Scale Modeling of the Failing Heart

**DOI:** 10.1371/journal.pone.0134869

**Published:** 2015-07-31

**Authors:** Elham Kayvanpour, Tommaso Mansi, Farbod Sedaghat-Hamedani, Ali Amr, Dominik Neumann, Bogdan Georgescu, Philipp Seegerer, Ali Kamen, Jan Haas, Karen S. Frese, Maria Irawati, Emil Wirsz, Vanessa King, Sebastian Buss, Derliz Mereles, Edgar Zitron, Andreas Keller, Hugo A. Katus, Dorin Comaniciu, Benjamin Meder

**Affiliations:** 1 Department of Medicine III, University of Heidelberg, Heidelberg, Germany; 2 DZHK (German Centre for Cardiovascular Research), Heidelberg, Germany; 3 Siemens Corporation, Corporate Technology, Imaging and Computer Vision, Princeton, New Jersey, United States of America; 4 Siemens AG, Corporate Technology, Erlangen, Germany; 5 Siemens Corporation, Corporate Technology, Sensor Technologies, Princeton, New Jersey, United States of America; 6 Biomarker Discovery Center Heidelberg, Heidelberg, Germany; 7 Department of Human Genetics, Saarland University, Homburg, Germany; 8 Klaus Tschira Institute for Computational Cardiology, Heidelberg, Germany; Medical University Innsbruck, AUSTRIA

## Abstract

**Background:**

Despite modern pharmacotherapy and advanced implantable cardiac devices, overall prognosis and quality of life of HF patients remain poor. This is in part due to insufficient patient stratification and lack of individualized therapy planning, resulting in less effective treatments and a significant number of non-responders.

**Methods and Results:**

State-of-the-art clinical phenotyping was acquired, including magnetic resonance imaging (MRI) and biomarker assessment. An individualized, multi-scale model of heart function covering cardiac anatomy, electrophysiology, biomechanics and hemodynamics was estimated using a robust framework. The model was computed on n=46 HF patients, showing for the first time that advanced multi-scale models can be fitted consistently on large cohorts. Novel multi-scale parameters derived from the model of all cases were analyzed and compared against clinical parameters, cardiac imaging, lab tests and survival scores to evaluate the explicative power of the model and its potential for better patient stratification. Model validation was pursued by comparing clinical parameters that were not used in the fitting process against model parameters.

**Conclusion:**

This paper illustrates how advanced multi-scale models can complement cardiovascular imaging and how they could be applied in patient care. Based on obtained results, it becomes conceivable that, after thorough validation, such heart failure models could be applied for patient management and therapy planning in the future, as we illustrate in one patient of our cohort who received CRT-D implantation.

## Introduction

Systolic HF is a leading cause of hospitalization in developed countries. Its prevalence is estimated to increase by 25% till 2030 [1], with an associated health care expenditure increase of 120% in the same period. HF can be the result of a variety of underlying conditions, including ischemic, genetic and inflammatory cardiomyopathy, hypertension, metabolic diseases or toxic injury [2, 3]. Currently, guideline-based therapies of HF rely mainly on the stage of the disease depending on patients’ symptoms, cardiac performance as assessed by imaging modalities, and few objective biomarkers, but rather neglect individual factors regarding disease etiology and prognosis. Although survival of systolic HF patients has improved over the past decades, morbidity and mortality rates are still unacceptably high. To improve survival, new clinical scores, molecular biomarkers and image-based technologies are required for more precise classification of disease stages and calculation of the individual risk [4].

The past decades have seen a tremendous research effort in the computational modeling of cardiac function [5–22]. While they were first developed for biology and disease understanding [5, 6, 8, 13, 23], these models are now maturing towards clinical application [5, 7, 10–12, 14, 22, 24–29]. This included the identification of the mathematical laws underlying biological mechanisms such as cardiac electrophysiology, biomechanics, or hemodynamics, all of which have been derived or verified using experimental data. Later, multi-scale mathematical frameworks have been developed to simulate the cardiac function at the whole-organ scale [6, 7, 9, 14, 15, 24, 30]. One challenge for a clinical use remained the issue of personalization from patients’ clinical images and data, which is now one of the main research interests of the scientific community (see for instance [7, 11, 14, 15, 22, 24, 31]). Hence, a large-scale evaluation is still missing due to the complexity of the personalization algorithms (from anatomy to function) and the required computational power of the models.

In this study, we developed a multi-scale model of HF and explored if it can provide new insights on HF patients in a sufficiently large population. In particular, computationally efficient models coupled with robust parameter estimation methods are employed to estimate an individualized model on 46 HF cases. We show for the first time that estimated model parameters from imaging are very consistent with cardiac imaging, lab tests and prognosis scores. Because the employed multi-scale computational model is predictive, we also illustrate in one case its potential in personalized planning of cardiac resynchronization therapy.

## Methods

### Clinical Workflow and Patient Recruitment

The present study has been approved by the ethics committee, medical faculty of Heidelberg and participants have given written informed consent. In this case-only study, 46 patients were included with diagnosis of non-ischemic systolic HF and underwent diagnostic coronary angiography, echocardiography, cMRI, comprehensive clinical phenotyping and biomarker measurements for the evaluation of a newly HF onset. The majority of patients also received invasive hemodynamic assessment (left circulation in 43 (93%) cases, right circulation in 37 (80%) cases). To capture all stages of systolic heart failure, we also included symptomatic patients with only slightly decreased systolic performance in the initial assessment of cardiac function by echocardiography or ventricular angiography (LV-EF <55%). All patients showed signs or symptoms of HF (e.g. breathlessness, reduced walking capacity, ankle swelling or pulmonary crackles) [3]. Patients with relevant CAD, valvular heart disease, acute myocarditis or a history of cardiotoxic chemotherapy were excluded.

### Patient-Specific Computational Model of Human Non-ischemic Systolic Heart Failure

**Fig 1:**
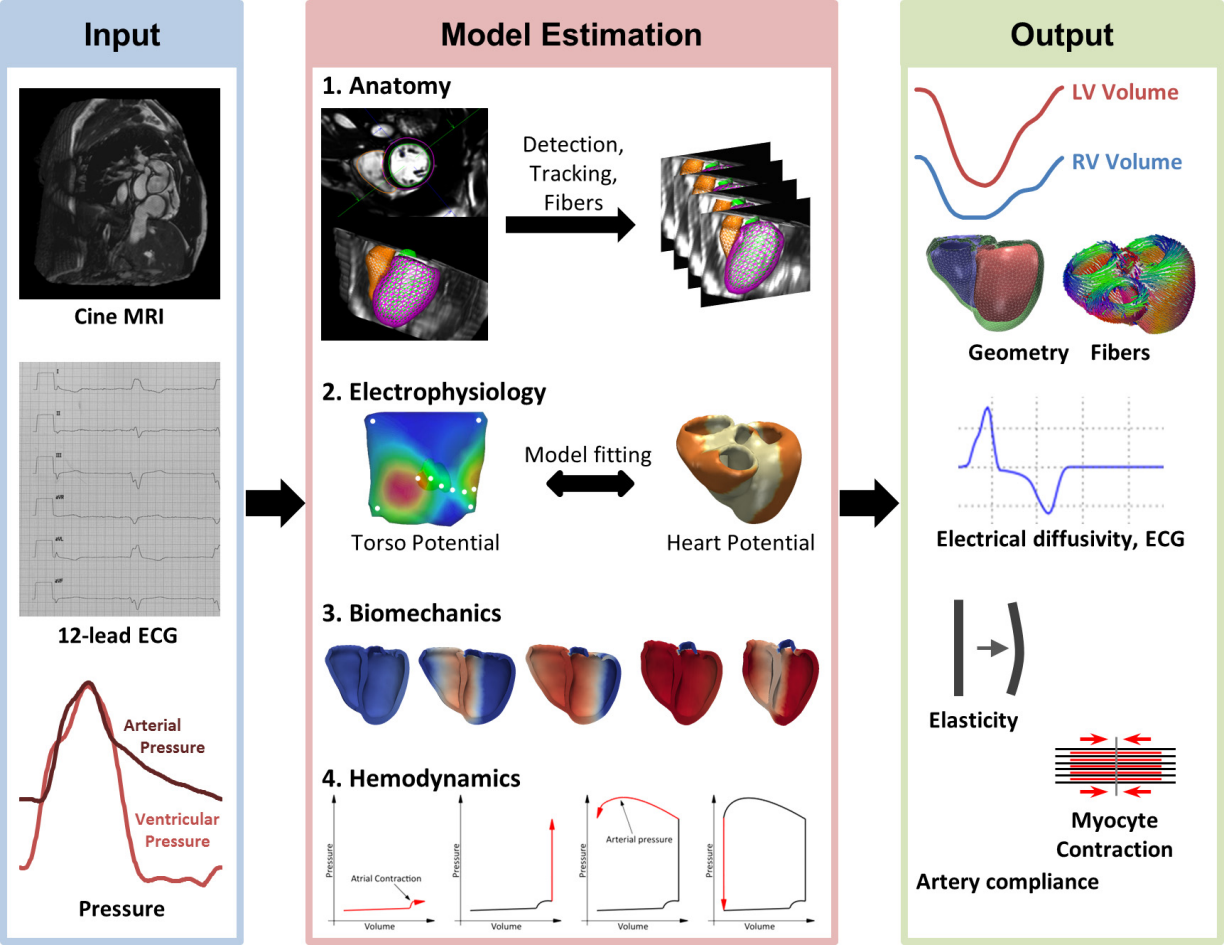
Overview of the modeling pipeline, from clinical data (input) to multi-scale, multi-physics cardiac models (output). The framework components are described in detail in methods.

For each patient of the cohort (n = 46), a multi-scale, multi-physics model of cardiac function was estimated based on the pseudonymized imaging data, 12-lead ECG and invasive pressure catheterization [31]. Heart anatomy [32], electrophysiology [22, 31], hemodynamics and biomechanics [31, 33] were modeled (Fig 1). Each of these components is personalized to fit the observed physiology of the patient [31]. An overview of the approach is described in this section. The reader is referred to the cited references for more details due to lack of space.

#### Anatomy

First a detailed model of the bi-ventricular myocardium is generated. The myocardium is segmented under expert guidance from short-axis cine cMRI. The model includes the left and right endocardia, LV outflow tract, RV inflow and outflow tracts, and LV epicardium. Each of these components is estimated consistently and simultaneously using a machine-learning approach [32] and verified by an expert. In brief, the algorithm is trained from a large database of annotated cine cMRI to automatically detect the heart position, rotation and scale in the image. Each step is learned using the Marginal Space Learning framework and Probabilistic Boosting Trees [32]. A boundary detector is also learned from the database to recognize myocardium contours in the images. On unseen data, the algorithm first uses the location detectors to find the heart at the end-diastole time frame, and then fits a mean heart shape model whose contours are finally deformed according to the boundary detectors to match the myocardium boundaries. Once the model is detected, it is tracked over time using a symmetric image registration algorithm [34]. An expert then carefully verifies the tracked contours and edits them interactively if needed. Blood pool volume is finally computed at each time instant from the anatomical model.

Next, the surfaces of each component at end diastole are fused to form a unified, volumetric model of the bi-ventricular myocardium. As the RV epicardium is hardly visible in the cMRI, a standard thickness of 3mm is applied. The surface of the volumetric model is labeled as LV (left endocardium), RV (right endocardium), LV septum and RV septum based on the original segmentation [14, 33]. Finally, a model of fiber architecture is generated following a rule-based approach [13]. In this study, LV elevation angles vary linearly from -60° to the epicardium to 0° to the mid-wall to 60° to the endocardium, RV elevation angles vary from -80° to 80°.

#### Electrophysiology

Patient cardiac electrophysiology (EP) is computed using a reaction-diffusion equation, so-called mono-domain equation, which models the dynamics of the trans-membrane potential and its diffusion throughout the myocardial tissue. In this study, the Mitchell and Schaeffer (M-S) model of the trans-membrane potential is employed [35]. Contrary to more complex ionic models, which consider the ionic interactions through a large number of transmembrane channels [36], the M-S model captures the bulk inflow and outflow ionic exchanges as described in [35]. Despite its simplified formulation, M-S model has shown good behavior in pathological situations, in particular alternans, arrhythmias and wave re-entries [15, 35, 37]. The M-S model is controlled by four parameters, which have a direct impact on the dynamics of the action potential and the restitution curve. In particular, the duration of the action potential is directly controlled by the parameter τc, which is related to the QT duration in the ECG. The other parameters (τopen, τin and τout), which control the diastolic interval and the restitution curve, are kept to their default values in this study [35]. The computed potential is then diffused throughout the myocardial tissue according to the electrical conductivity, tissue anisotropy defined by the cardiac fibers and cardiac activation pathways [22, 37]. The electrical signal starts at the LV and RV septum (His bundle). A thin, 1mm-thick sub-endocardial layer is defined to mimic the Purkinje system, with two different electrical conductivities for the left and right ventricle Purkinje systems (c_LV_ and c_RV_ respectively). The rest of the myocardium is modeled with a slower electrical conductivity c_Myo_ [22]. To cope with tissue anisotropy, the electrical conductivity is assumed to be nine times higher along the fibers (equivalent to a fiber speed three times faster than cross-fiber). The model is solved on the patient heart anatomy by using a novel framework based on the Lattice-Boltzmann method (LBM-EP) [37], which allows near real-time computations. Finally, torso potentials are calculated using the boundary element methods (BEM) as described in [22], from which 12-lead ECG signals are calculated and displayed to the user. To cope with the lack of full-torso images, a patient-specific torso model was obtained as follows. A torso atlas was first registered to the cMRI scout images acquired in axial, sagittal and coronal views. The heart of the torso atlas was rigidly aligned with the heart of the patient. The torso surface was then deformed using an affine transformation to match the patient torso boundaries visible in the scouting images [22]. ECG leads were then placed at their clinical locations on the individualized torso model. In [22], the properties of the coupled heart-torso EP model were extensively investigated and validated against numerical experiments, while computational efficiency and scalability was explored in [38].

The personalization of the EP model is performed in three steps, as described in details in [39]. First, myocardium conductivity (c_Myo_) is automatically estimated using inverse modeling. To this end, the NEWUOA algorithm [40], a gradient-free optimization method, is used such that computed QRS duration (cQRSd) matches the mQRSd measured in the ECG signal. Next, LV and RV subendocardial conductivity, c_LA_ and c_RV_, are estimated to capture the measured electrical axis (EA). Finally, the value of the parameter τc is directly inferred from the QT interval. These three steps are iterated until convergence is reached (generally after two or three iterations). This fitting procedure is done only once per patient: at the end of it, the current state of patient cardiac electrophysiology is captured.

#### Biomechanics

The cardiac motion resulting from the electrophysiology computed as previously described is calculated according to a multi-scale biomechanical model of the cardiac tissue [31, 33]. More precisely, a two-element Hill model is employed. The passive properties of the myocardium are modeled as isotropic linear elastic tissue. A co-rotational implementation is employed to correctly capture the large deformations and rotations during systole [41]. Tissue elasticity is parameterized by the Young’s modulus E and estimated from the images as described below. Incompressibility is ensured by setting the Poisson ratio υ to 0.48 for all cases in this study. A phenomenological model of the active force generated during systole is coupled to the passive model [14, 42]. The force is timed according to the electrophysiology while its maximal amplitude is controlled by the maximum active force parameter σ. The force increases and decreases according to an exponential law with two different decay parameters, k_ATP_ and k_RS_, related to ATP binding and myosin head release rates [42]. The heart is fixed in space using a contact-based pericardium force, defined as the epicardial bag at end-diastole [43], and a basis stiffness constraint, modeled as springs attached around each of the four cardiac valves to mimic the ventricular attachment to the artery and atria. Both parameters E and σ can be set regionally. In particular, LV E and σ are estimated automatically for each patient by minimizing the sum of the squared differences between computed and measured ejection fraction (EF), stroke volume (SV), end-diastolic volume (EDV), end-systolic volume (ESV), end-diastolic pressure (EDP) and end-systolic pressure (ESP). As for the EP component, the minimization is performed using NEWUOA optimizer, as described in [31, 40], and done only once for each case.

#### Hemodynamics

Finally, the biomechanical model is coupled with a lumped model of intra-cardiac hemodynamics to capture the pre-load and post-load conditions of the patients [14, 23, 33]. The four cardiac phases are calculated (filling, isovolumetric contraction, ejection, isovolumetric relaxation). A well-validated 3-element Windkessel model is employed to model the arteries [44], whose parameters (artery compliance C, peripheral resistance Rp, characteristic resistance Rc and remote pressure pr) are estimated from the arterial pressure catheterization measurement and the MRI-based blood flow measurement using an optimization approach [31]. The lumped model of atrial contraction presented in [23] is also used. The ventricular pressure is calculated such that the isovolumetric constraint is ensured through a prediction-correction scheme as described in [33]. The phases are fully controlled by the electrophysiology (which triggers cardiac contraction) and hemodynamics load. In this study, no circulatory system was used contrary to [23], the two ventricles were not hemodynamically connected between each other. Although this could be a limitation to capture adaptation to pulmonary or systemic hypertension for instance, such a simplification showed promising results in heart failure modeling and cardiac resynchronization therapy, as highlighted by recent results reported by other groups [7, 14]. The complete biomechanical model is implemented in SOFA, an open-source simulation framework [45].

On a standard desktop machine (Intel Xeon E5629, 2.40GHz, NVidia GeForce GTX580 card, 4GB RAM), segmenting the chambers takes from 2–3s to 2–3 minutes per frame depending on the level of editing required, building the anatomical model takes 1 minute (including meshing, tagging and fibers, all steps being automatic), the forward EP computation takes 5s, the forward EM computation takes 3 minutes. The personalization procedure, which requires several forward model estimations, takes between 3 to 4 hours per patient. Overall, going from the clinical data of a patient to a complete, personalized model takes approximately 3–5 hours. At each step, the user can verify the result and validate them before moving to the next step.

### CRT simulation

In addition to the main contribution of this study, which is the population analysis, we also illustrate in this manuscript how such a multi-scale model could be used for cardiac resynchronization therapy planning and guidance in our cohort. The model estimation procedure described in the previous section has been designed such that the model matches the observed patient physiology. However, the estimated parameters may not be unique due to noise and sparsity in the data, and model assumptions, as quantified in [31, 46] for instance. Another way to evaluate the model is to test its predictive power after a change in the cardiac system. Indeed, even if two sets of parameters would yield the same visible cardiac motion, it is likely that only one would enable precise prediction of system changes, due to therapy for instance. We illustrate the predictive power of our framework in one patient of our cohort, who underwent CRT-D device implantation due to LV-EF<35%, left bundle branch blockage (QRSd = 124 ms) and HF NYHA class III despite optimal medical therapy. An additional 12-lead ECG recording was acquired one day after device implantation and during testing of three different stimulation protocols: biventricular pacing (BiV; LV-RV delay = 20 ms), RV only stimulation and DDT-40 LV triggered. In all three cases we acquired comprehensive echocardiography. We first fitted the model to the baseline ECG (cQRSd = 123 ms (measured = 124 ms) and EA = -17° (measured = -17°), S5A Fig). The estimated electrical conductivities were equal to 320 mm^2^/s, 1600 mm^2^/s and 2200 mm^2^/s for the myocardium, LV endocardium and RV endocardium, respectively. It should be noted that in this experiment the estimation aimed to capture T-wave onset timing only, but not T-wave morphology. We then reproduced the CRT on the virtual heart based on the interventional report and the applied protocols (S5B Fig).

### Statistical Analysis

Statistical analyses were carried out with „R”software (version 3.0.2). Histograms were calculated using the “hist” function with standard parameters. Outliers were detected with the Grubbs test available in the “outliers” package. Correlation coefficients were assessed by the “cor” function and corresponding significance values by the “cor.test” procedure. In order to visualize the output, customized scatter plots were generated. Smoothing of scatter plots was carried out by the “smoothScatter” function included in the bioconductor “geneplotter” package. On top and right of each scatter plot, the distribution of both plotted variables was respectively drawn as box plot.

## Results

### Personalized multi-scale computer models are detailed representations of systolic HF

In this study, we consecutively recruited patients subjected to invasive and imaging-based diagnosis of HF. Table 1 summarizes the clinical characteristics of this study cohort, while Table 2 provides the findings of ECG and cMRI recordings. After data anonymization and curation, we transferred imaging and hemodynamic data to the developed modeling framework.

**Table 1:** Clinical characteristics of the patient cohort with non-ischemic systolic HF.

Age, mean (SD), y	52.34 (14.9)
BMI, mean (SD), kg/m²	26.7 (4.5)
Male sex, No. (%)	38 (70.4)
Diabetes, No. (%)	6 (13.3)
Smoker, No. (%)	28 (60.0)
Alcohol excess, No. (%)	3 (6.7)
Family history of DCM, No. (%)	18 (24.3)
Lipid profile	
Total cholesterol, mean (SD), mg/dl	174 (36)
High-density lipoprotein, mean (SD), mg/dl	45 (13)
Low-density lipoprotein, mean (SD), mg/dl	100 (32)
Triglyceride, mean (SD), mg/dl	147 (168)
White blood cell count, mean (SD), /nl	8.1 (2.6)
NT-proBNP, mean (SD), ng/l	2789 (4486)
hs-TNT, mean (SD), pg/ml	13.6 (14.5)
Heart rate, mean (SD), beats/min	70 (17)
Blood pressure, mean (SD), mm Hg	
Systolic	121 (18)
Diastolic	76 (12)
NYHA functional class, No. (%)	
I	9 (19.6)
II	23 (50.0)
III	13 (28.3)
IV	1 (2.2)
Medications at baseline, No. (%)	
Aspirin	14 (17.9)
ß-Blocker	45 (95.7)
ACE inhibitor or ARB	46 (100)
Aldosterone antagonist	27 (41.5)
Other diuretics	26 (39.4)
Warfarin	15 (19.5)
Statin	21 (29.6)
Digoxin	6 (7.0)

ACE: angiotensin-converting enzyme; ARB: angiotensin II receptor blocker; DCM: dilated cardiomyopathy; No: Number; NYHA: New York Heart Association; SD: Standard deviation.

**Table 2:** ECG and cMRI findings of patient cohort with non-ischemic systolic HF.

LV-EF, mean (SD), %	36 (14)
< 30, No. (%), %	16 (34.8)
30–44, No. (%), %	17 (37.0)
45–54, No. (%), %	10 (21.7)
≥ 55, No. (%), %	3 (6.5)
LV-SV, mean (SD), ml	88.5 (23.6)
QRS duration, mean (SD), ms	118 (27)
QT duration, mean (SD), ms	406 (42)
EA, mean (SD)	13 (59)

EA: electric axis of the heart; LV-EF: left ventricular ejection fraction; LV-SV: left ventricular stroke volume; No: Number; SD: Standard deviation.

#### Anatomy Model

**Fig 2:**
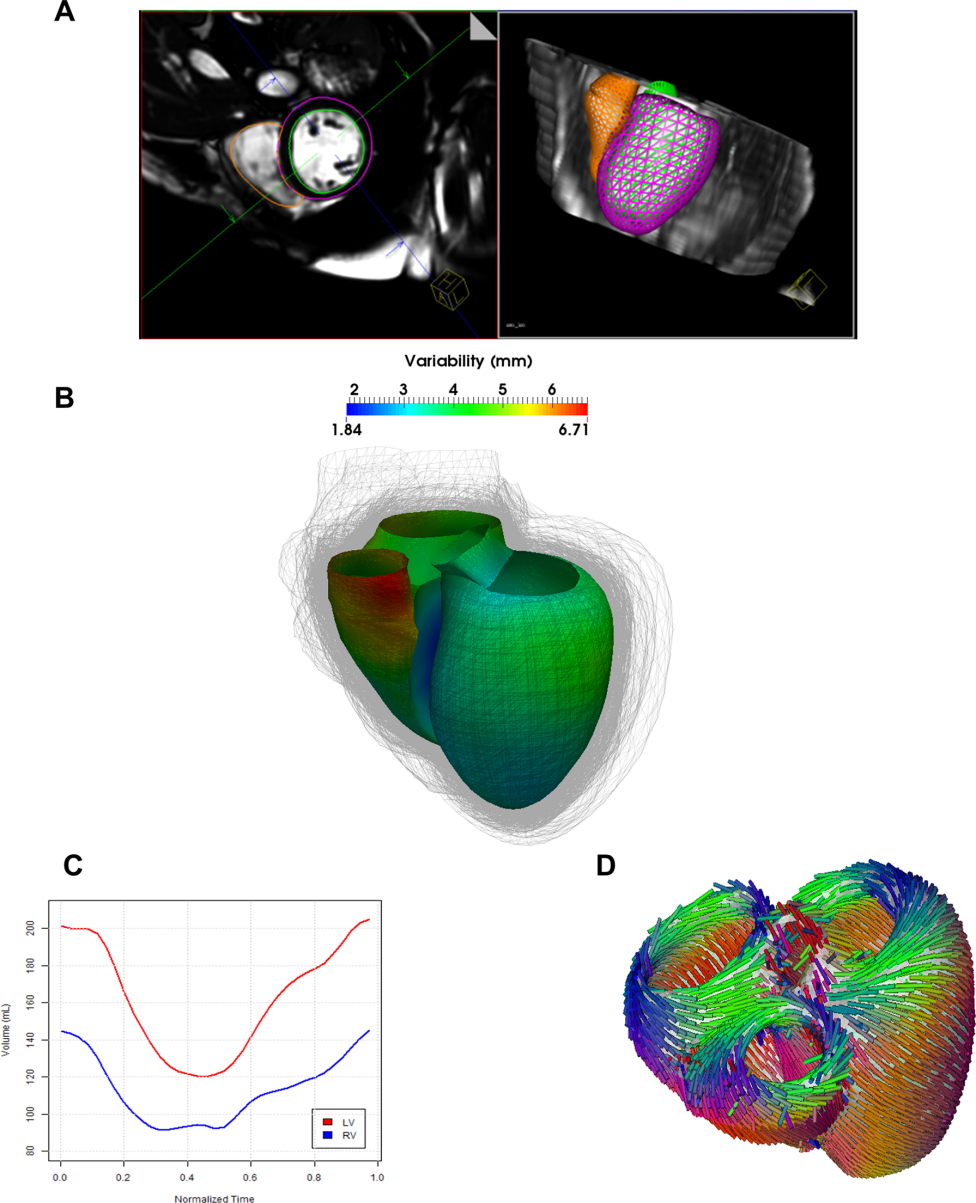
Automated estimation of the 3D anatomical model. **A)** Automatic segmentation of the right and left ventricle. **B)** Observed variability in cardiac anatomy (shape is color-coded on a template) estimated from the HF cohort. The representation indicates the variability in phenotypes from the cohort. **C)** After the different steps of the model computation are finished, computed intracardiac volume variations can be estimated. **D)** Fiber architecture applied to the personalized heart models.

Three dimensional models of the bi-ventricular anatomy were generated from cMRI under expert guidance (Fig 2A). A considerable variability of bi-ventricular shapes could be observed across the population, with an average variation of about 5mm around a mean atlas calculated from the population [47] (Fig 2B). After heart tracking and volume computation (Fig 2C), mean left ventricle ejection fraction (LV-EF) of the computed models was 36 +/- 14%. In particular, three patients were identified having LV-EF in the normal range (≥ 55%), 10 mild reduction in LV-EF, 17 moderate LV-EF decrease and 16 had a severe dysfunction. Fig 2D shows the fiber architecture computed on the anatomical model in a typical case [30].

#### Electrophysiology Model

**Fig 3:**
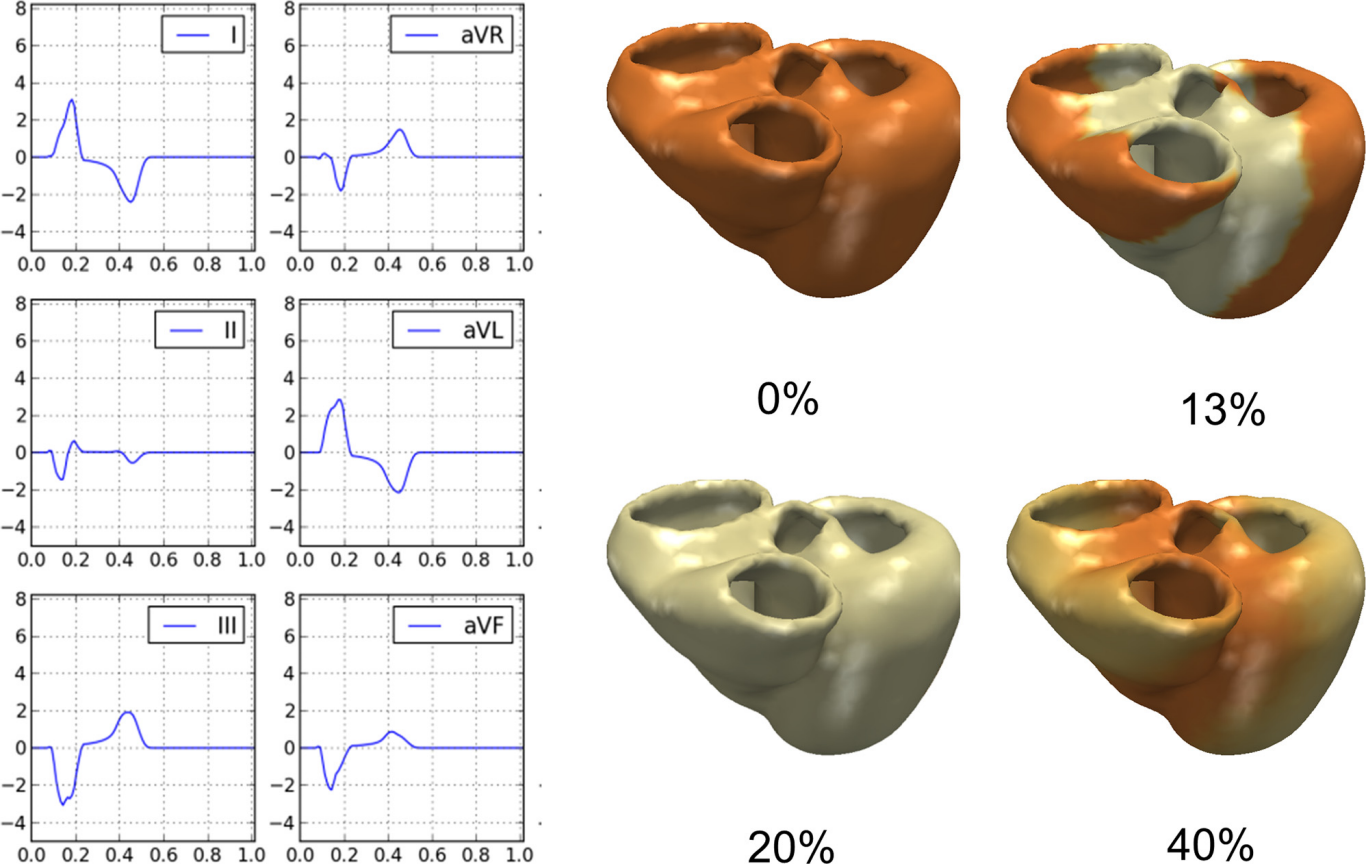
Patient-specific electrophysiology computation. **Left Panels:** Computed ECG traces from the model in the patient exemplarily chosen. **Right Panels:** Computed trans-membrane potential propagation throughout the cardiac cycle (time in % of cycle length).

Electrical conductivity for the myocardium (c_Myo_), left (c_LV_) and right endocardia (c_RV_) were estimated based on the anatomical model and the QRS duration (QRSd) and electrical axis (EA) measured from the 12-lead ECG traces [31]. After personalization, computed cQRSd matched the measured mQRSd within 12 +/- 10 ms accuracy (R = 0.82, p<10^−7^), confirming the ability of the model to capture HF electrophysiology and the efficacy of the ECG personalization algorithm. The EA was in some cases more difficult to estimate (mean error = -23 +/- 55 degrees, R = 0.32, p<0.05), probably due to localized pathologies (e.g. bundle blocks) that was not captured by our global personalization procedure. The distribution of the estimated conductivities across the population is displayed in S1 Fig. Fig 3 shows the electrical activation and the computed ECG traces (first six leads) calculated for the same patient illustrated in Fig 1. For that example, computed QRSd, QTd and EA were equal to 172 ms, 458 ms and -42 degrees respectively, compared to the measured values of 175 ms (ΔQRS = 3ms), 456 ms (ΔQT = 2ms) and -42° (ΔEA = 0°) of the surface 12-lead ECG.

#### Hemodynamics Model

**Fig 4:**
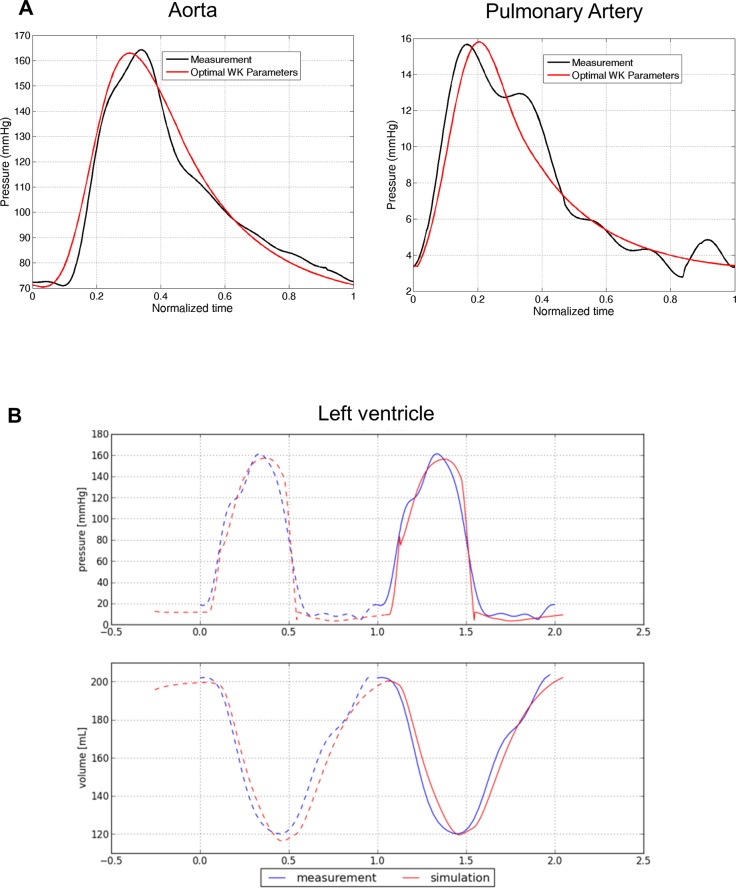
Patient-specific hemodynamics. **A)** Personalized computation of arterial flow after personalization of the Windkessel parameters. **B)** Measured and computed LV pressure and volume curve in one patient, showing the high concordance between the clinical and modeling data.

Patient-specific artery compliance and resistances were estimated from cMRI-derived volume curves and invasive pressure catheterization [31]. Fig 4A illustrates, again in the same case, the computed arterial pressure curves in the left (systemic) and right (pulmonary) main arteries after personalization. As one can see, the model captured the main trends of the pressure dynamics, in particular systolic and diastolic pressures. Table 3 reports descriptive statistics of the estimated artery compliance (C), peripheral resistance (Rp), characteristic resistance (Rc) and remote pressure (Pr) (histograms reported in S2 Fig). It should be noted that for the very few patients who did not have invasive pressure measurements, generic values were applied (Table A in S1 File).

**Table 3:** Statistics of estimated Windkessel parameters throughout the studied population.

	C (mm^3^/mmHg)	Rp (mmHg/mm^3^)	Rc (mmHg/mm^3^)	Pr (mmHg)
**Aorta**	2269±1375	3.64e-4±2.27e-4	4.72e-5±2.46e-5	52±23
**Pulmonary artery**	2757±1869	1.51e-4±1.51e-4	2.91e-5±2.26e-5	15±9

#### Biomechanical Model

Finally, we computed cardiac electromechanics for all cases. LV stiffness E and maximum active force σ were estimated (S3 Fig) [31]. The model allowed us also to derive the time-varying ventricular volume and pressure, which we compared with the invasive measurements to further evaluate model goodness of fit (Fig 4B). Qualitatively, the fitting was judged by two independent experts to be excellent in 29 patients (i.e. perfect match of the volume and pressure curves up to the noise level), good in 15 (i.e. the main trends of the volume and pressure curves were captured, but not in all details), and sub-optimal in 2 cases (curves did not match). Quantitatively, S4 Fig shows the correlation between the cMRI-derived and model-derived LV-EF (A) and stroke volume (SV) (B), underlining the precision of the HF model in terms of global cardiac function.

### Estimation of novel cardiac functional parameters using a virtual failing heart

One advantage of the multi-scale model is that it provides functional parameters that cannot be directly measured from image analysis or clinical phenotyping, potentially providing novel surrogates of HF and further insights into patient’s condition or disease stage. Table B in S1 File summarizes some of the new parameters derived from the model. To examine the clinical value of those new parameters, we performed correlation analyses (Pearson coefficient) with rich clinical phenotypes. To avoid bias in the study, only the data that was not used in the model fitting procedure were investigated.

As shown in Table 4, a good correlation was found between left ventricular active force and the systolic blood pressure after physical activity. Left ventricular active force negatively correlated with the diagnosis and prognosis biomarker NT-ProBNP. Although these correlations are still modest, they provide a first hint at the potential use in further stratifying patients, especially since they may provide unambiguous disease information.

**Table 4:** Correlations between LV active force and LV stiffness and clinical presentations of patients.

	NT-proBNP(ng/l)	Heart rate at rest(/min)	Systolic Blood Pressureat rest (mmHg)	Systolic Blood Pressureafter physical activity (mmHg)
**LV-σ**	R = -0.5p = 2e-3	R = -0.5p = 1.2e-2	R = 0.5p = 4e-3	R = 0.5p = 7e-3
**LV-E**	R = 0.03p = 8e-1	R = -0.3p = 8e-2	R = 0.1p = 7.2e-1	R = 0.0p = 9.9e-1

**Fig 5:**
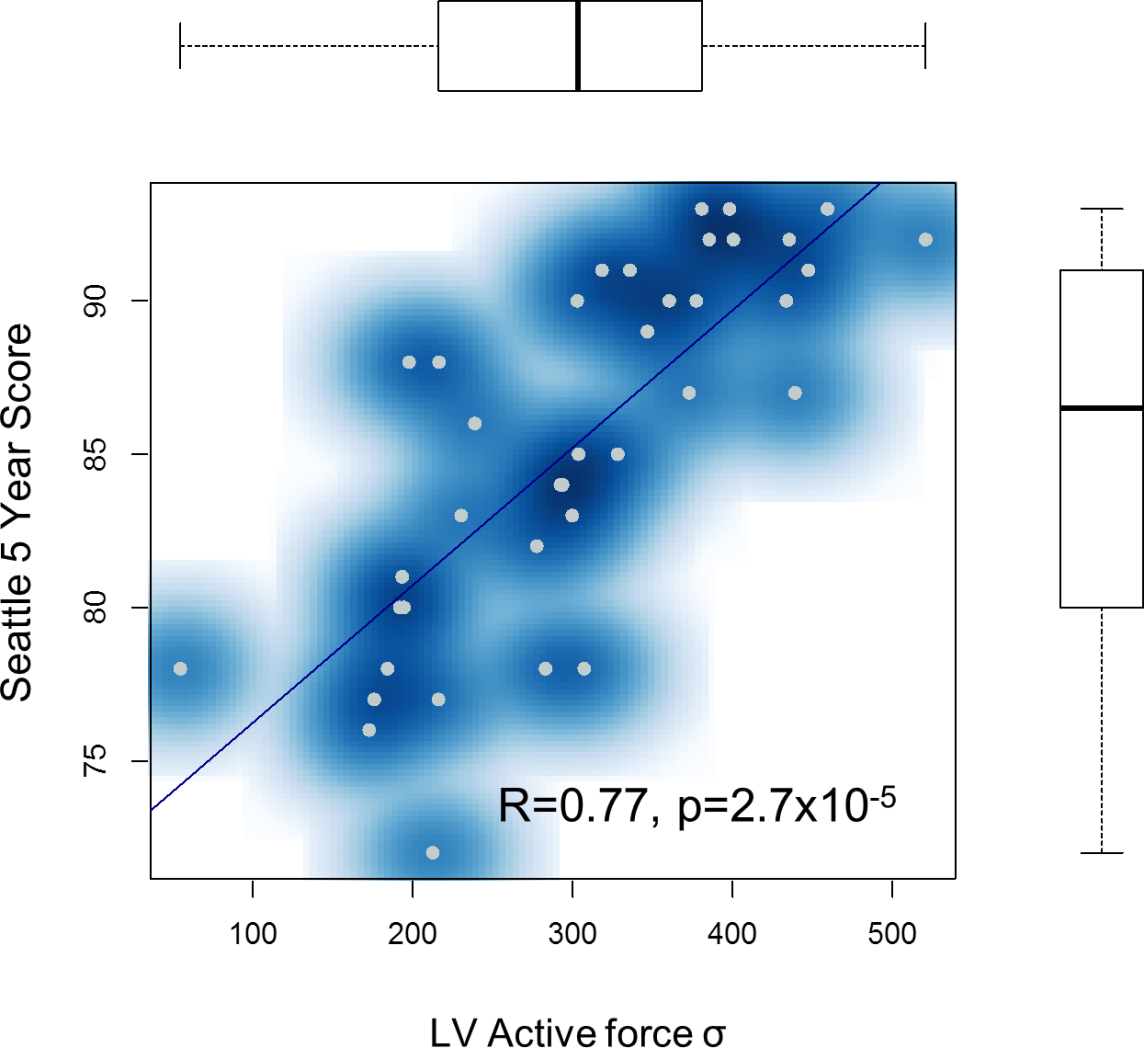
Correlation between Left ventricular active force and estimated outcome of the patients. Correlation plot showing the left ventricular active force in the patients (x-axis) and their Seattle 5 Year Score (y-axis).

To further explore a potential prognostic value, we performed correlation analyses of the unique parameters with the Seattle HF Score [48]. The Seattle HF Model predicts survival of HF patients by using a complex set of clinical characteristics such as the NYHA class, ischemic etiology, diuretic dose, left ventricular ejection fraction, systolic blood pressure, sodium, hemoglobin, percent lymphocytes, uric acid, and cholesterol. The Seattle score could be completely calculated in 40 patients at the time of their inclusion. After performing Grubb’s testing, one of 40 patients was detected as a statistical outlier and hence was excluded from the analysis. As shown in Fig 5, a highly significant correlation between LV active force σ and the Seattle 5-year score was found (R = 0.77, p = 2.7x10^-5^). Interestingly, this finding suggests that the maximum stress the myocardium can generate is predictive of HF outcome, the more impaired the myocardium contractility, the poorer the outcome.

### Computed heart models for therapy planning

As an example of application for personalized therapy, we simulated CRT using our virtual heart in one patient of our cohort. As shown in S5C Fig and Table C in S1 File, the model was able to successfully and highly accurately predict the changes in cardiac electrophysiology for different CRT stimulation protocols. It was able to predict the worsening of cardiac EP for both BiV and RV pacing modes (increased QRSd as a predictor of nonresponse [49, 50]) and also the preservation of EP function with the LV-triggered protocol. The later protocol was chosen by the independent treating electrophysiologist (independently from the model result), the patient showed 7 months after device implantation a significant improvement of clinical symptoms (NYHA III improved to NYHA II) as well as LV-EF (EF improved from 20% to 30%).

## Discussion

Thanks to advanced guideline-adherent pharmaco- and device therapy of HF, mortality and hospitalization have been reduced. Yet, HF is still one of the major causes of death and societal burden, requiring novel therapies and personalized diagnostics [51]. In particular, the impact of individual, patient-specific factors is largely neglected in current therapies. The addition of personalization to the treatments recommended in current guidelines is seen as an opportunity to further improve the outcome and quality of care in HF.

Besides the search for novel drug targets and HF biomarkers, the computational modeling community has been striving to provide novel solutions in HF care in the form of generative models of heart physiology [5, 7, 10–12, 14, 15, 22, 36]. The simulation of subcellular mechanisms is already possible with high fidelity compared to experimental data [30, 36, 52]. At the organ level, these simulations are far more complex, but have resulted in the development of prototypes of virtual hearts. Recent effort is now focusing on the translation of these models to the clinics [7, 14, 15]. The model presented in this study relies on validated biophysical models and numerical methods [22, 33] and combines this with a fitting framework that allowed for the first time investigation of an adequately sized cohort of clinically well-characterized HF patients [31]. Indeed, the experiments performed in this study highlighted the necessity of having computationally efficient numerical methods and streamlined, fully-integrated personalization procedures to minimize user interaction and enable efficient and reproducible workflows.

As shown in [22], the personalization approach is able to estimate one solution of the inverse problem, with a good fit to the data. We could statistically compare the estimated parameters with clinical phenotyping and imaging, thus verifying the clinical meaningfulness of these parameters. For instance, we could find that the myocardium active force, i.e. the ability of the myocardium to contract, could be an indicator of disease severity, similar to what was reported in [53] using a study on three cases.

As shown in [54], several sets of parameters could yield the same observable physiology and noise in clinical data can considerably decrease model precision. One advantage of the present study is the availability of extended phenotyping, including MRI, echocardiography, pressure catheterization and lab tests. This unique dataset helped decreasing parameter uncertainty [31], which is reflected by the highly reproducible estimation of cardiac performance when compared to measured parameters. Similarly, the sparsity of the clinical observation (12-lead ECG) yields to a significant level of uncertainty in estimated electrical conductivities, as quantified in [22]. Despite this limitation, the model was still able to predict therapy in the illustrative example reported in the results section and in [39]. The topic of parameter uncertainty is an important research area [25, 31, 46, 54], which requires further investigations.

Over the whole cohort of 46 patients, the correlation of the computed parameters to the values obtained through detailed clinical phenotyping reached promising statistical power, rendering it reasonable to derive additional parameters of cardiac function that are not directly assessable from imaging data. Given the available model, we could provide first estimates of new patient-specific parameters such as electrical conductivity, myocardial stiffness or cardiac active force in a HF cohort, which could be used in subsequent studies. While they may show some overlap with existing measures of HF severity, indicated by their correlation e.g. to natriuretic peptides, they also may provide unambiguous information about the state of an individual patient and his prognosis. Nominating these parameters as biomarker candidates, it will be interesting to validate them in independent cohorts and compare them to existing scores and measures, as we did with the Seattle HF score.

More thorough validation of predictive mathematical models of disease processes will be the key for their implementation into clinical practice. One approach is the evaluation of the predictive power of these models in terms of therapy outcome, such as cardiac resynchronization therapy (CRT). Although not the main focus of this study, we illustrate how such a model could be used for CRT planning, suggesting good predictive power in terms of acute QRS duration. From preoperative data, we calculated acute postoperative ECG and successfully compared the result with the clinical observation. Computational modeling for CRT planning has been investigated in the past [7, 12, 14, 26]. Yet, they were based on generic geometries [26], assumed the postoperative electrophysiology [14] or did not predict the resulting ECG while assuming postoperative hemodynamics [9]. Instead, in this manuscript we illustrate how cardiac electrophysiology and ECG could be predicted based on preoperative data only. Future works include a comprehensive evaluation of this aspect on a larger cohort.

Several assumptions were made in the model used in this study, which can be a limitation. First, the ECG computation assumed homogeneous tissue between the heart and the body surface, with one electrical conductivity [22]. While this simplification can have an impact on the shape of the ECG trace, numerical studies have shown that global parameters such as QRS and QT duration are minimally dependent on tissue heterogeneity and therefore justify our approach [55]. After personalization, the model precisely captured the main features of the heart’s function, such as QRS duration and QT intervals. EA was more difficult to capture. A potential reason for this limitation is the fact that cardiac electrophysiology was only globally personalized, thus missing potential localized pathological features like lines of blocks, as confirmed in recent experiments [39]. Another simplification of the model is the linear assumption of the myocardium tissue. Ex-vivo studies showed that cardiac tissue is an orthotropic, hyper-elastic material [56]. Future work includes using more advanced models including orthotropic tissue material and whole-body circulatory models for more accurate estimation of tissue properties and more realistic cardiac motion computation. First results in that direction were reported in [33, 54]. The biomechanical model was also globally fitted to the data. While this approach enabled capturing global parameters like EF and SV, regional impairments were not captured, contrary to [53]. In these cases, the global parameters might be biased to cope with the regional pathological feature.

## Supporting Information

S1 Fig Distribution of estimated electrical conductivity in the cohort. **A)** In average the myocardial electrical conductivity c_Myo_ was equal to 413 +/- 232 mm2/s. **B and C)** Left and right endocardial conductivity were higher as expected owing to the fast Purkinje conduction system.
(TIFF)

S2 Fig Distribution of estimated Windkessel parameters in the cohort. Upper panels: Hemodynamics features of the aorta. Lower panels: Hemodynamics features of pulmonary artery. For values see also Table A in S1 File.
(TIF)

S3 Fig Distributions of the estimated stiffness E and active force σ in the cohort. Average LV stiffness E is 590 ± 135 kPa and LV maximum active force σ is 295 ± 100 kPa in study population. These parameters can only be simulated and not directly measured from image sequences or clinical data.
(TIF)

S4 Fig Correlation between measured cardiac values from cMRI and computed ones. **A)** x: computed LV-EF, y: measured LV-EF from cMRI. The distribution of both plotted variables is respectively drawn as box plot. **B)** x: computed LV-SV, y: measured LV-SV from cMRI.
(TIF)

S5 Fig Computed heart models for prediction of CRT therapy. **A)** Computed ECG trace (thick blue line) overlaid on measured ECG trace before CRT implantation. As one can see, the model was able to capture the main features of the ECG trace precisely. It should be noted that it was not in the scope to capture T-wave morphology (see text for details). **B)** Position of the CRT leads (in green) placed on the model according to lead positions derived from orthogonal chest x-rays. **C)** Observed and predicted (thick blue line) ECG traces for three tested stimulation protocols. The model was able to qualitatively predict EP CRT response.
(TIF)

S1 File **Table A in S1 File.** Default Windkessel parameter values used when pressure data was not available, see text for details. **Table B in S1 File.** Parameters estimated using the multi-scale heart model. **Table C in S1 File.** Observed and predicted ECG parameters for different CRT protocols.
(DOC)
