# Supplementary figures and images for: Towards Personalized Cardiology: Multi-Scale Modeling of the Failing Heart
Source: PLoS One. 2015 Jul 31;10(7):e0134869. doi: 10.1371/journal.pone.0134869 (PMC4521877; doi:10.1371/journal.pone.0134869)

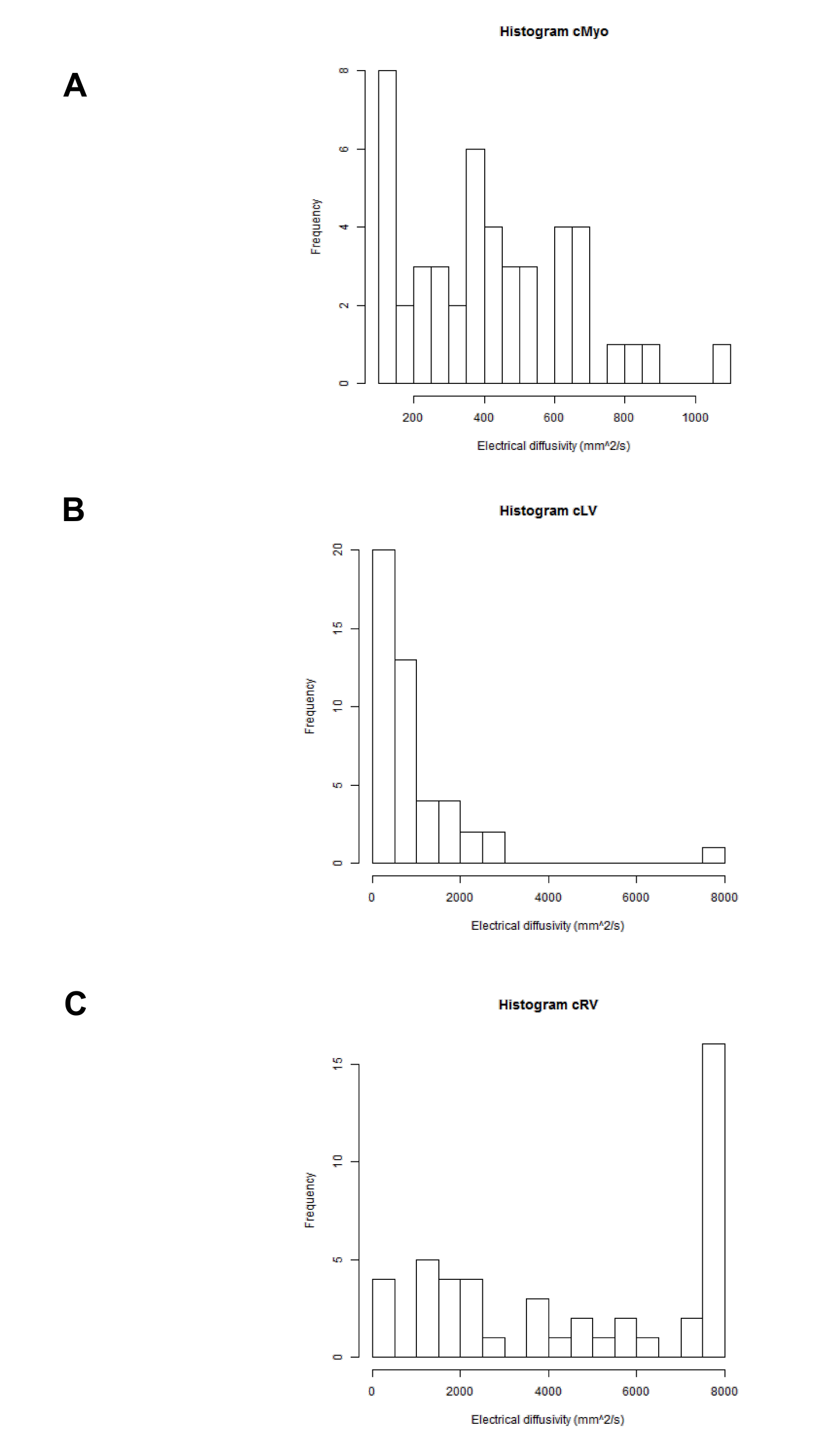

Supplement: S1 Fig — A) In average the myocardial electrical conductivity cMyo was equal to 413 +/- 232 mm2/s. B and C) Left and right endocardial conductivity were higher as expected owing to the fast Purkinje conduction system. (TIFF) [file pone.0134869.s001.tiff]

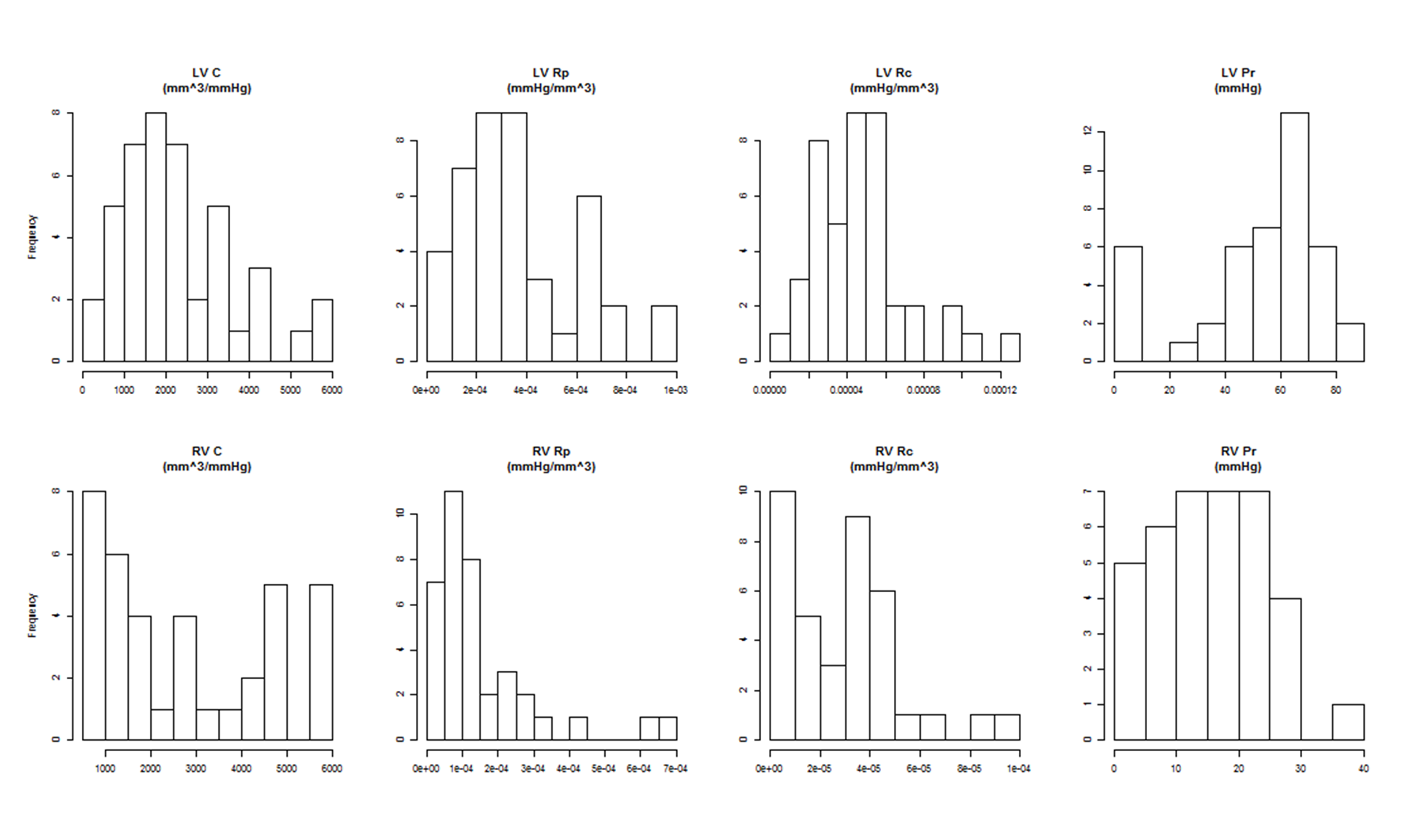

Supplement: S2 Fig — Upper panels: Hemodynamics features of the aorta. Lower panels: Hemodynamics features of pulmonary artery. For values see also Table A in S1 File. (TIF) [file pone.0134869.s002.TIF]

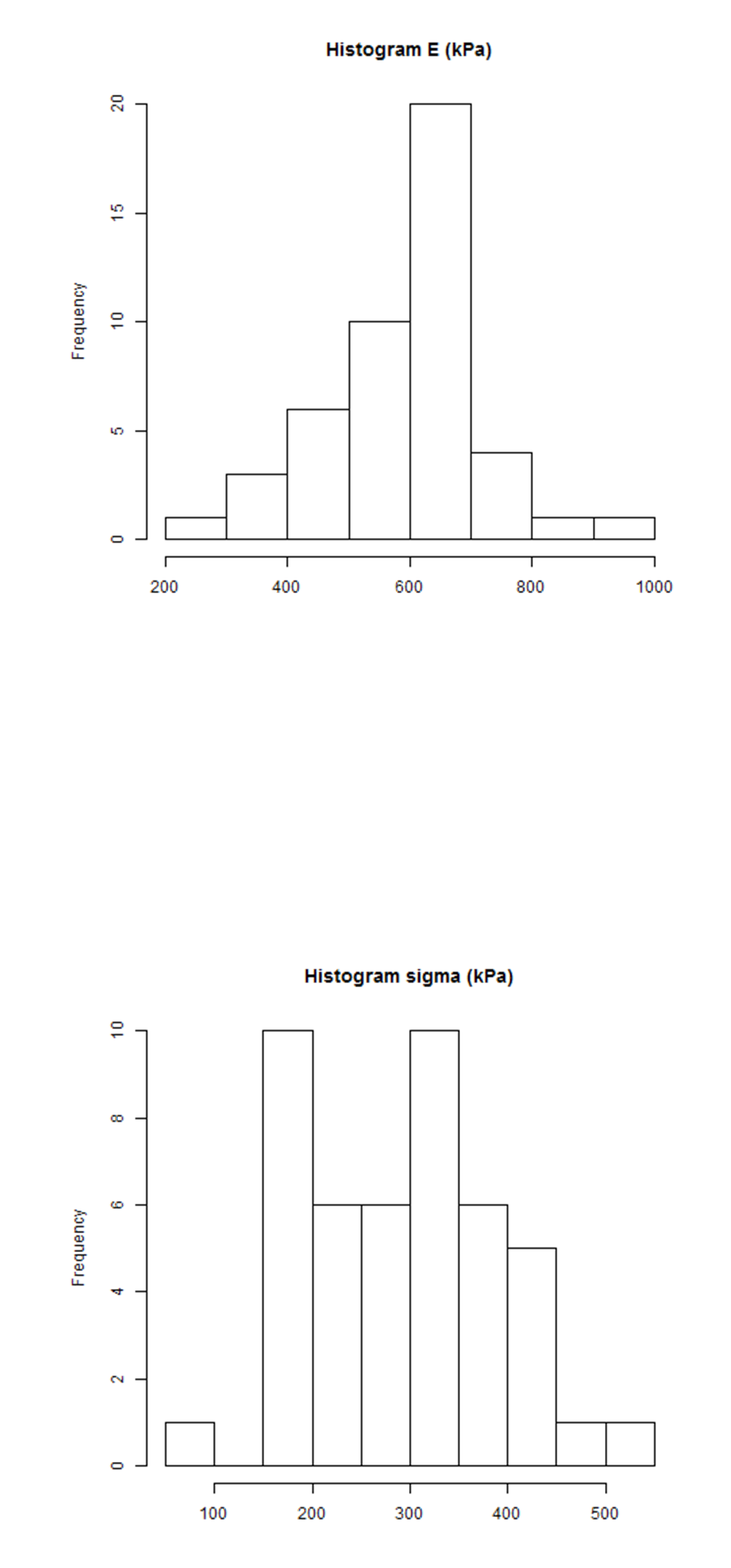

Supplement: S3 Fig — Average LV stiffness E is 590 ± 135 kPa and LV maximum active force σ is 295 ± 100 kPa in study population. These parameters can only be simulated and not directly measured from image sequences or clinical data. (TIF) [file pone.0134869.s003.TIF]

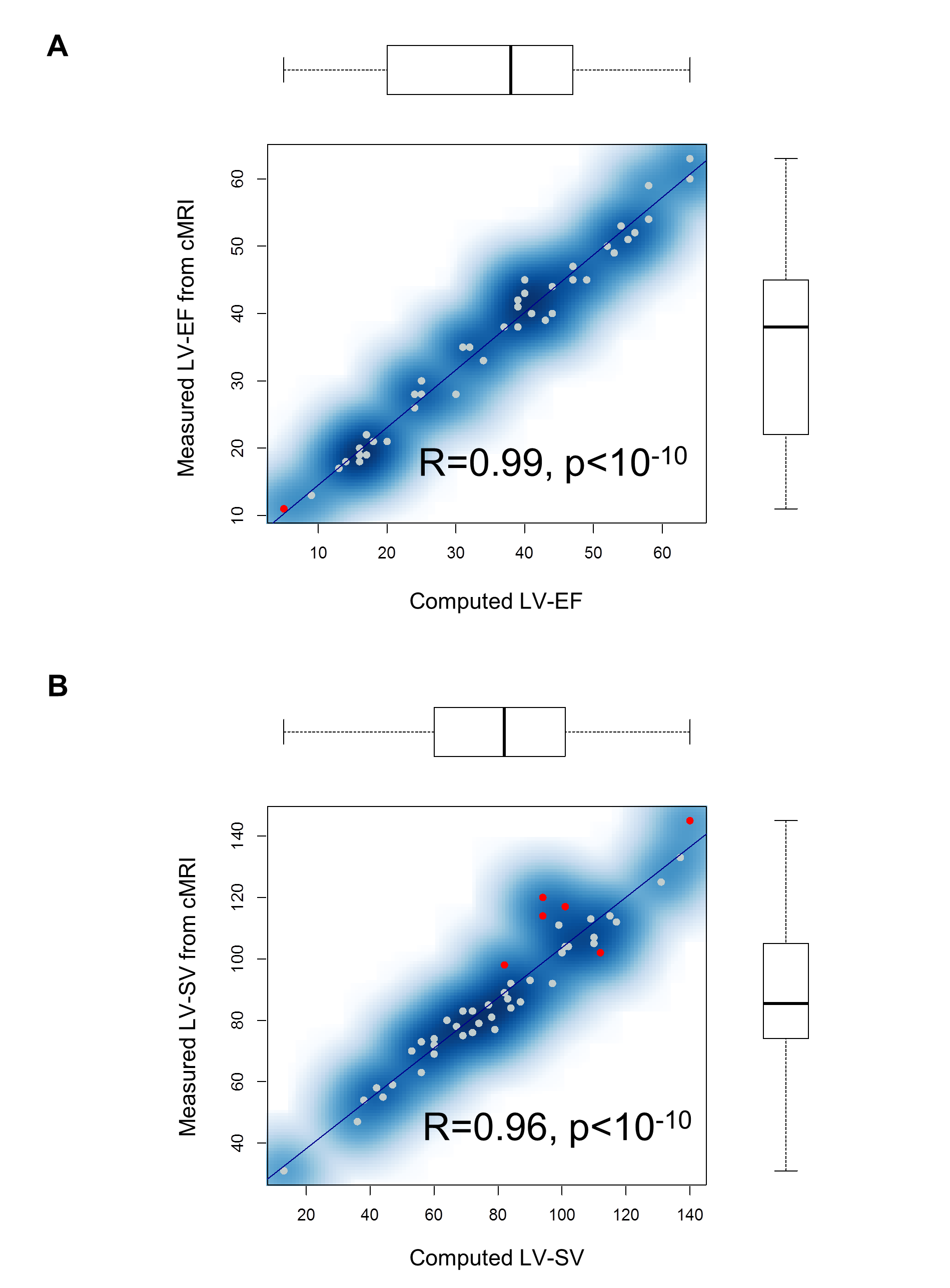

Supplement: S4 Fig — A) x: computed LV-EF, y: measured LV-EF from cMRI. The distribution of both plotted variables is respectively drawn as box plot. B) x: computed LV-SV, y: measured LV-SV from cMRI. (TIF) [file pone.0134869.s004.TIF]

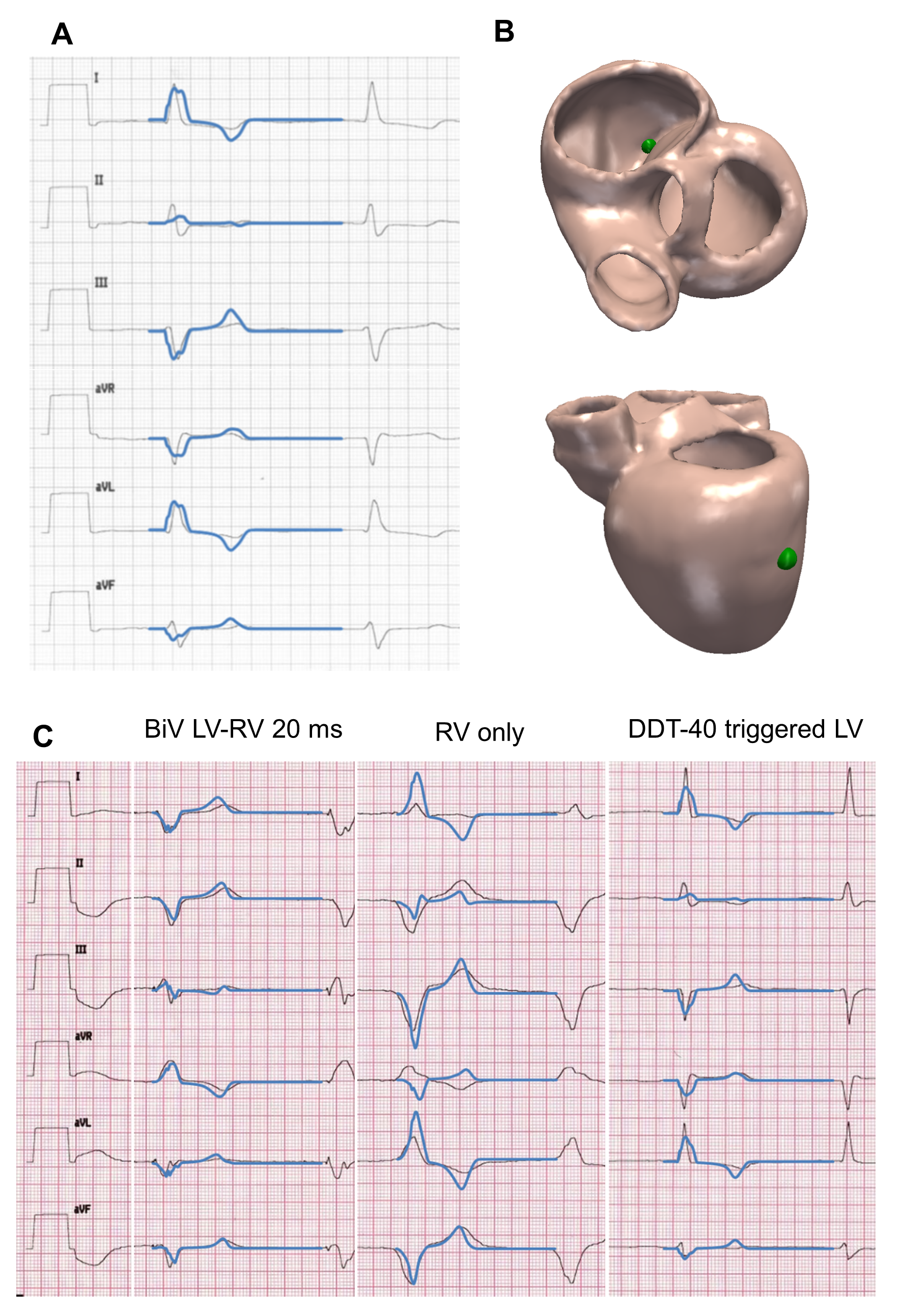

Supplement: S5 Fig — A) Computed ECG trace (thick blue line) overlaid on measured ECG trace before CRT implantation. As one can see, the model was able to capture the main features of the ECG trace precisely. It should be noted that it was not in the scope to capture T-wave morphology (see text for details). B) Position of the CRT leads (in green) placed on the model according to lead positions derived from orthogonal chest x-rays. C) Observed and predicted (thick blue line) ECG traces for three tested stimulation protocols. The model was able to qualitatively predict EP CRT response. (TIF) [file pone.0134869.s005.TIF]
