# Supplementary material for: Towards Personalized Cardiology: Multi-Scale Modeling of the Failing Heart
Source: PLoS One. 2015 Jul 31;10(7):e0134869. doi: 10.1371/journal.pone.0134869 (PMC4521877; doi:10.1371/journal.pone.0134869)
Supplement: S1 File — Table A in S1 File. Default Windkessel parameter values used when pressure data was not available, see text for details. Table B in S1 File. Parameters estimated using the multi-scale heart model. Table C in S1 File. Observed and predicted ECG parameters for different CRT protocols. (DOC) [file pone.0134869.s006.doc]

Table A: Default Windkessel parameter values used when pressure data was not available, see text for details

|  | **C (mm^3^/mmHg)** | **Rp (mmHg/mm^3^)** | **Rc (mmHg/mm^3^)** | **Pr (mmHg)** |
| --- | --- | --- | --- | --- |
| **Aorta** | 1600 | 5e-4 | 3e-5 | 60 |
| **Pulmonary artery** | 2100 | 4e-4 | 1.5e-5 | 4 |

Table B: Parameters estimated using the multi-scale heart model

| Anatomical Features | | Unit |
| --- | --- | --- |
|  | Ejection fraction (LV-EF and RV-EF) | % |
|  | Stroke volume (LV-SV and RV-SV) | mL |
| Electrophysiology Features | | |
|  | Electrical conductivity (myocardium, LV endocardium, RV endocardium) | mm^2^/s |
| Biomechanics Features | | |
|  | LV stiffness (E) | kPa |
|  | LV active force (σ) | kPa |
| Hemodynamics Features (aorta, pulmonary artery) | | |
|  | Artery compliance | mm^3^/mmHg |
|  | Peripheral resistance | mmHg/mm^3^ |
|  | Characteristic resistance | mmHg/mm^3^ |
|  | Remote pressure | mmHg |

**Table C:** Observed and predicted ECG parameters for different CRT protocols.

|  | **QRS duration (ms)** | | **Electrical axis (degrees)** | |
| --- | --- | --- | --- | --- |
|  | **Measured** | **Predicted** | **Measured** | **Predicted** |
| **BiV LV-RV 20ms** | 171 | 171 | 161 | 215 |
| **RV only** | 184 | 191 | -69 | -38 |
| **DDT-40 triggered LV** | 125 | 105 | -16 | -18 |
